# Investigation of canine visceral leishmaniasis in a non-endemic area in Brazil and the comparison of serological and molecular diagnostic tests

**DOI:** 10.1590/0037-8682-0182-2021

**Published:** 2021-09-06

**Authors:** Anaiá da Paixão Sevá, Ana Pérola Drulla Brandão, Silvia Neri Godoy, Rodrigo Martins Soares, Helio Langoni, Bruna Cristine Rodrigues, Mariana Zanchetta e Gava, Paula Ferraz de Camargo Zanotto, Tatiana Jimenez-Villegas, Roberto Hiramoto, Fernando Ferreira

**Affiliations:** 1 Universidade de São Paulo, Departamento de Veterinária Preventiva e Saúde Animal, São Paulo, SP, Brasil.; 2 Universidade Estadual de Santa Cruz, Departamento de Ciências Agrárias e Ambientais, Ilhéus, BA, Brasil.; 3 Instituto Chico Mendez de Conservação da Biodiversidade, São Sebastião, SP, Brasil.; 4 Universidade Estadual Paulista “Júlio de Mesquita Filho”, Departamento de Higiene Veterinária e Saúde Pública, Botucatu, SP, Brasil.; 5 Instituto Adolfo Lutz, São Paulo, SP, Brasil.

**Keywords:** Seroprevalence, DPP^®^-Dual Path platform, IFA, ELISA, Dog, *Cãoservação* Program

## Abstract

**INTRODUCTION:**

Visceral leishmaniasis (VL) is an important zoonosis in Brazil. Previous identification of parasitized dogs can also help prevent the disease in humans, even in non-endemic areas of the country. The Brazilian Ministry of Health recommends diagnosis in dogs using a DPP^®^ (rapid test) as a screening test and an immunoenzymatic assay (ELISA) as a confirmatory test (DPP^®^+ELISA), and culling infected dogs as a legal control measure. However, the accuracy of these serological tests has been questioned.

**METHODS:**

VL in dogs was investigated in a non-endemic area of the São Paulo state for three consecutive years, and the performances of different diagnostic tests were compared.

**RESULTS:**

A total of 331 dog samples were collected in 2015, 373 in 2016, and 347 in 2017. The seroprevalence by DPP^®^+ELISA was 3.3, 3.2, and 0.3%, respectively, and by indirect immunofluorescence assay (IFA), it was 3.0, 5.6, and 5.5%, respectively. ELISA confirmed 18.4% of DPP^®^ positive samples. The concordance between the IFA and DPP^®^ was 83.9%. The concordance between IFA and DPP^®^+ELISA was 92.9%. A molecular diagnostic test (PCR) was performed in 63.2% of the seropositive samples, all of which were negative.

**CONCLUSIONS:**

In non-endemic areas, diagnostic tests in dogs should be carefully evaluated to avoid false results.

## INTRODUCTION

Visceral leishmaniasis (VL) is an important zoonosis worldwide. In South America and Brazil, VL is expanding geographically with an increase in human cases, becoming a great challenge to public health^1^ ^-^ ^3^. In Brazil, this disease is caused by *Leishmania infantum*, and the vectors involved in its transmission are sandflies of the species *Lutzomyia longipalpis* ^4^, *Lu cruzi* ^5^, *Migonemyia migonei* ^6^, and *Pintomyia fischer* ^7^. 

In addition to humans, this disease can affect both domestic and wild species^8^. In Brazil, infected dogs constitute the main domestic reservoir of the parasite, once among other reasons is a great source of infection for vectors, thus playing an important role in VL transmission to humans^9^. Therefore, previous identification of parasitized dogs can help prevent and control the disease in dogs and humans, including in non-endemic areas of the country. In São Paulo State, VL in humans and dogs have been spreading mainly from the northwest to the southeast of the state. Over the years, the dispersion of the disease in dogs has occurred before the identification of human cases^3^. 

The Brazilian Ministry of Health, in its VL Control and Surveillance Program (VLCSP), recommends identification and culling of infected dogs as one of the legal instruments to control the disease^10^. Parasitological techniques are considered the reference standard to identify infected animals^11^, but serological tests can be used as a tool in epidemiological surveys to facilitate diagnosis and decision making for euthanasia^10^. For diagnosis in dogs, the tests recommended by VLCSP are the rapid test, dual-path, chromatographic immunoassay (dual-path platform - DPP^®^) as a screening test, and the enzyme-linked immunosorbent assay (ELISA) as a confirmatory test^10^. However, all serological tests have been extensively questioned, especially regarding their accuracy^12^ ^-^ ^15^. 

Biological samples from a domestic dog population were collected for three consecutive years to evaluate the diagnoses of VL in dogs in a non-endemic area. To date, there have been no dog or human reported cases of VL in the area or nearby municipalities. In addition, the present investigation was used to compare the performance of the tests in such scenarios.

## METHODS

### Study area

**FIGURE 1:**
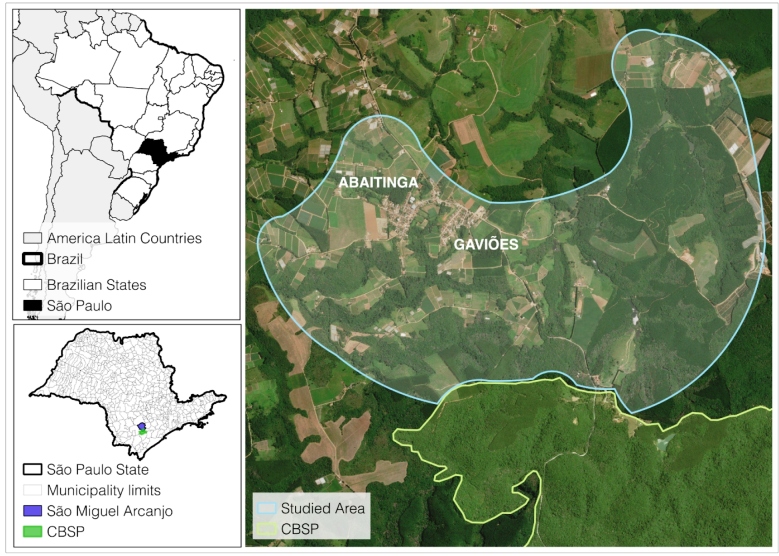
Neighborhoods of Abaitinga and Gaviões where residences were sampled in the São Miguel Arcanjo municipality of São Paulo, Brazil. **CBSP:** Carlos Botelho State Park.

Dog samples were collected in two neighborhoods of São Miguel Arcanjo Municipality, namely Abaitinga and Gaviões, within São Paulo State, Brazil (Figure 1). These areas are located in the surroundings of Carlos Botelho State Park (CBSP), a Brazilian Conservation Unit, inside a large remnant of the Atlantic Rain Forest. Most dogs living in the area have free access to streets and the Park. According to the Surveillance Epidemiological Center of the State (CVE-SP), these areas are considered free of VL transmission. Areas with VL transmission in the state of São Paulo are shown in Supplementary Material Figure 1. 

### Data collection

Three census surveys were carried out in April 2015, 2016, and 2017 (1^st^, 2^nd^, and 3^rd^ collections, respectively), covering all residences in the study area and sampling those with dogs. If residents were not at home, the visit was repeated at least three times on subsequent days. If dog owners refused to participate in the survey, their animals were not included in the study. During the household visits, the owner of the dog signed a “free and informed consent form”, which was approved by the Ethics Commission of Animal Use of the School of Veterinary Medicine and Animal Science of the University of São Paulo (CEUA/FMVZ/USP) under protocol number CEUA 2452231014. 

After obtaining authorization from the owner, the household was georeferenced, and blood samples of dogs over 3 months of age were collected from the jugular, cephalic, or femoral veins. These samples were stored at 5ºC for a maximum of 5 h until serum was extracted by centrifugation at 1500 rpm for 10 min for later serological evaluation of antibodies anti-*L. infatum* reactions*.*

Conjunctival swab samples were collected in the 1^st^ and 3^rd^ collections, and popliteal lymph node samples were collected by fine-needle aspiration in the 2^nd^ collection. Such sampling was done together with blood collection, and depended on favorable operational issues, such as animal behavior and owner approval. The samples were homogenized in water for storage in microtubes (2ml) until molecular diagnostic testing.

All samples (serum blood, diluted lymph node cells, and conjunctival swabs) were stored at -20°C for further analysis.

### Laboratory analysis

Serum samples were submitted to the two serological tests recommended by VLCSP. All samples were tested by the screening test (DPP^®^ Canine VL, Biomanguinhos FIOCRUZ) for detection of antibodies against K26/K39 of amastigotes of *Leishmania*. Then, samples found to be positive in the screening test were verified by the confirmatory test (immunoenzymatic assay canine VL, Biomanguinhos, FIOCRUZ - ELISA) to detect antibodies against soluble antigens of promastigotes, using recombinant A2 protein, which is currently considered an amastigote-specific protein or the best used *Leishmania* antigen. Both tests were performed at the Adolfo Lutz Institute in São Paulo Municipality.

All samples were also tested for antibodies against *Leishmania* spp. using the indirect immunofluorescent assay test (IFA)^16^, employing *L. major*-like antigens, adopting 40 as the cut-off titer^10^. Samples with positive results were titrated until the final titer was obtained. Positive and negative control serum samples were obtained from dogs from endemic areas of São Paulo State (Botucatu municipality and region).

### Molecular diagnostic analysis

This analysis was performed only in samples of animals that tested positive by at least one serological test. qPCR was performed to amplify a 120-base-pair fragment of the *Leishmania* kinetoplast minicircle DNA (kdna)^17^. Samples tested positive by qPCR were confirmed with conventional nested PCR based on primers directed to ITS1, as described by Schönian et al.^18^. The resulting ITS1 products were sequenced bidirectionally using the forward and reverse primers using the Sangers dideoxynucleotide method and the ABI PRISM BigDye Terminator kit (Applied Biosystems) following the manufacturer’s protocol. 

### Concordance analysis

The agreement of diagnostic tests, such as that between RIFI and DPP^®^ and between RIFI and DPP^®^ followed by ELISA (DPP^®^+ELISA), was obtained according to an alternative *Kappa* coefficient (modified Kappa)^19^. This alternative method is recommended to solve limitations, such as prevalence close to extremes (0 and 100%) and/or asymmetric and imperfectly unbalanced contingency tables^19^, which is the case in the present study, in non-endemic areas, with low prevalence. The proportions of positive and negative concordance were calculated according to the method of Thrusfield^20^. Both analyses were performed using Microsoft Excel^®^ software.

Maps were performed using QGIS^®^ version 3.8.

## RESULTS

**FIGURE 2:**
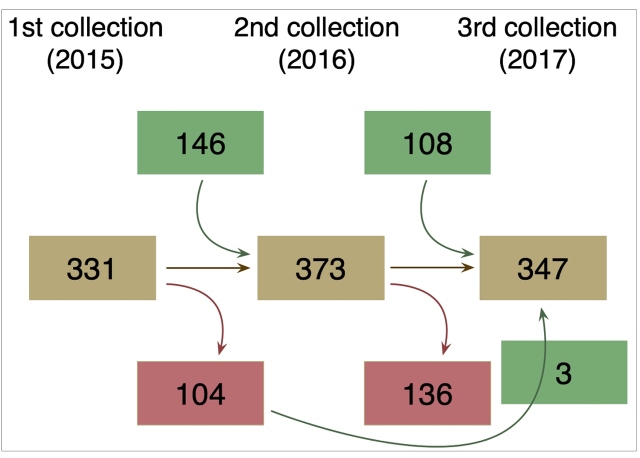
Dog population dynamics in the study area, according to the years of study. Number inside the boxes indicated the number of dogs. Total number of sampled dogs (brown boxes); dogs that were not recollected due to deaths or exits (red boxes); and new dogs included due to births or entries (green boxes).

In 2015, 2016, and 2017, there were 189, 200, and 179 domiciles with dogs, respectively. The number of animals sampled was 331, 373, and 347, respectively, totaling 1,051 samples. In all surveys, only three owners refused to participate. Of the total households, 75% had an area less than 300 m^2^ and were close to or in areas where the streets were paved (termed houses); 20% were rural properties larger than 300 m^2^ (termed country houses); and the last 5% were small properties located on an agricultural farm (termed farmhouses). In the 2^nd^ and 3^rd^ sampling, some animals were not available for recollection due to their death or exit from the study area, which also affected the variations in the number of domiciles sampled in the three years. Three animals were sampled only in the 1^st^ and 3^rd^ sampling because the owners were absent during the 2^nd^ visit. New dogs were included in the study due to birth or entry into the study area (Figure 2). The number of animals sampled with recollection in the three years of the study was 157.

The prevalence of DPP^®^ positive samples in 2015, 2016, and 2017 was 12.9 (43/331), 12.6 (47/373), and 6.9% (24/347), respectively, with an average of 10.8%. 

**FIGURE 3:**
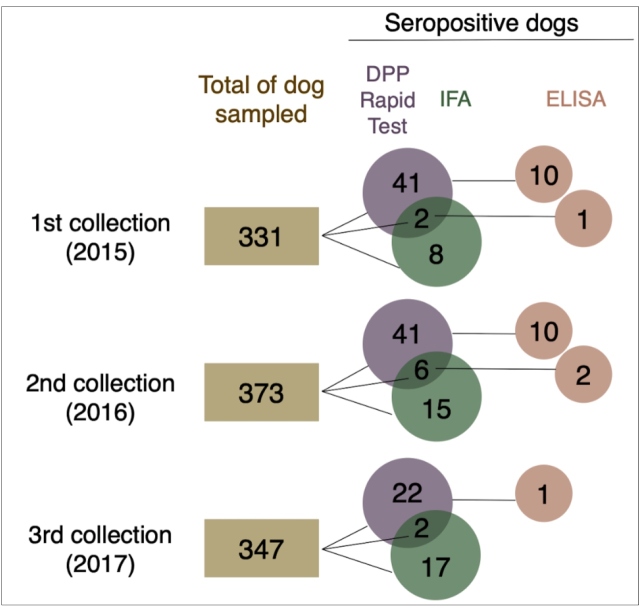
The number of dogs sampled (brown boxes) and seropositivity for DPP (Rapid Test - purple circles), IFA (Immunofluorescent Assay - green circles), and ELISA (Immunoenzymatic Assay - red circles), according to the 1st, 2nd, and 3rd collection.

ELISA confirmed, on average, 18.4% (24/114) of DPP^®^ positive samples, being 25.6 (11/43), 25.5 (12/47), and 4.2% (1/24) in the 1^st^, 2^nd^, and 3^rd^ collections, respectively (Figure 3). 

Considering the criterion adopted by the Brazilian Ministry of Health, in which an animal is considered infected if DPP^®^ and ELISA tests are positive, the prevalence in each survey was 3.3, 3.2, and 0.3%, respectively. This represents an average prevalence of 2.5%. The incidence in the 2^nd^ and 3^rd^ collections was equal to the prevalence since there were no animals positive for both tests in two subsequent years. One animal remained DPP^®^ positive in all three surveys. Four animals were positive only by DPP^®^ in the first two years, and two animals were positive for DPP^®^ in the 1^st^ and 3^rd^ collections.

The seroprevalence by IFA was 4.7%, on average, being 3.0, 5.6, and 5.5% in each survey, respectively (Figure 3). The most frequent serum titers were 40 and 80 (both with 36%), followed by 160 (14%), 320 (6%), and 640 (8%). Only one animal was positive by IFA in two consecutive years, presenting titers of 40 and 160 in the 1^st^ and 2^nd^ collections, respectively. One dog presented positive reactions in the 1^st^ and 3^rd^ collections, with titers of 40 and 160, respectively.

In the 1^st^ collection, the IFA titers of seropositive samples varied from 40 to 160, with 40 being the most frequent. In the 2^nd^ and 3^rd^ collections, the IFA titers varied from 40 to 640, with 80 being the most frequent. However, there was no significant statistical difference between the years in terms of titers. Considering the samples with higher IFA titers (three samples with a titer of 320 and two samples with a titer of 640), only one of them (titer of 320) was found positive with the DPP^®^ screening test.

IFA and DPP^®^ tests presented a concordance (modified Kappa) of 83.9%, and the proportion of positive and negative concordances were 0.11 and 0.92, respectively. However, the highest concordance (modified Kappa = 92.9%) was observed between IFA and DPP^®^+ELISA, and the proportion of positive and negative concordances were 0.08 and 0.93, respectively. These data were calculated according to the results presented in Table 1.

**TABLE 1:** Distribution of positive and negative samples, according to diagnostic methods.

		DPP			DPP + ELISA		Total
		pos	neg		pos	Neg	
IFA	pos	10	40		3	47	50
	neg	104	897		21	980	1,001
Total		114	937		24	1,027	1,051

**FIGURE 4:**
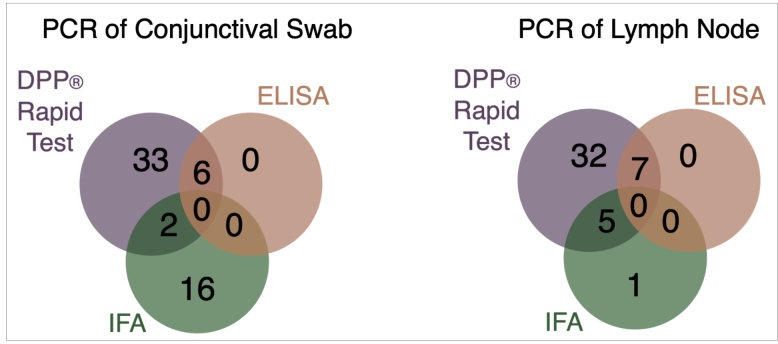
The number of seropositive samples for DPP (Rapid Test - purple circles), IFA (Immunofluorescent Assay - green circles), and ELISA (Immunoenzymatic Assay - red circles), who were tested by PCR of conjunctival swabs and lymph nodes in all samples.

PCR tests were performed in 22, 47, and 33 samples from the 1^st^, 2^nd^, and 3^rd^ collections, respectively, representing 63.2% (102/154) of all seropositive samples. Molecular analyses were performed on 57 conjunctival swabs (CS) and 45 lymph node (LN) samples (Figure 4). All samples were negative in both the molecular tests (qPCR and conventional nested PCR). Among the positive samples for DPP^®^, IFA, and DPP^®^+ELISA, 74.6 (85/114), 48.0 (24/50), and 54.2% (13/24) were tested by PCR. Of the five samples with higher titers of IFA (three with a titer of 320 and two with a titer of 640), only one sample (of CS) was tested by PCR, with a titer of 320. 

## DISCUSSION

The performance of ELISA as a confirmatory test for positive samples by DPP^®^ was different from that of other studies. Riboldi et al. reported that 2.9% of the dogs were DPP^®^+, and ELISA confirmed 16.6% of them^21^. ELISA confirmation was reported to be higher in other studies, such as up to 58.6% in 13.8% of DPP^®^ confirmed prevalence^14^ and 42.8% in 83.3% DPP^®^ confirmed prevalence (50/60)^22^.

It is important to reinforce that these two kinds of biological samples (CS and LN) used for molecular diagnostic testing are considered adequate to diagnose the parasite in infected dogs. Aschar et al. studied an endemic area, and among the 92 dogs included in the study, 11 were seropositive for DPP^®^+ELISA, with 54.4 (6), 63.6 (7), and 36.4% (4) of them being positive with the molecular diagnosis of CS, LN, and both CS+LN, respectively^23^. In their study, symptomatic and asymptomatic animals were positive for molecular diagnostics, but the prevalence of each was not specified^23^. Riboldi et al. (2018) observed a low concordance of PCR in seropositive animals for DPP^®^+ELISA, as evidenced by the low density of parasites^21^. According to Lopes et al. (2017), PCR applied to CS samples has proven to be a promising approach for the diagnosis of VL in dogs, when compared with PCR of LN and blood and serological tests (ELISA and DPP^®^) in an endemic area of São Paulo state^12^. Lombardo et al. found that 24.5% (40/163) and 22.1% (36/163) of the infected animals were positive by qPCR of LN and CS samples, respectively^24^. However, this study was performed in an endemic area in Europe^24^. In addition, they observed a significant association between the unhealthy clinical status and the overall molecular positive samples^24^.

Although not all DPP^®^-positive samples have been tested by molecular analyses, the unanimous negative results in molecular analyses warn of the possibility of the DPP^®^ average prevalence (10.8%) being cross-reactions or false positive results. This is because the test has low specificity and cross-reactions have been described. The specificity is lower in low-endemic areas (59.4%)^15^ than in high-endemic areas (95.1%), with a sensitivity of 90.6%^25^. Grimaldi et al. and Porrozzi et al. found a DPP^®^ specificity of 98%^26^ ^,^ ^27^, however, they also identified cross-reactions with *Leishmania braziliensis*, which causes cutaneous leishmaniasis in Brazil.

Some human cases of cutaneous leishmaniasis have been reported in the region of the studied area (Supplementary Material Figure 2)^28^. However, the fact that we did not have a reaction to this parasite species by PCR in seropositive animals does not mean the absence of the disease in the municipality. In addition, PCR was not performed for all seropositive samples. 

The specificity of DPP^®^ was higher in the absence of clinical signs. Fiqueiredo et al. found a specificity of 72.9% for asymptomatic animals and 56.4% for symptomatic animals^15^, and Grimaldi et al. found a specificity of 96.0% in asymptomatic dogs^26^. This test specificity can be high for the study area since there were no animals with classical clinical symptoms of the disease, as described by Ciaramella et al. (1997)^29^. When both tests (DPP^®^+ELISA) were used serially, the specificity increased compared to using them alone, reaching 98.9%^25^. 

According to Alvar et al. (1994), the “*gold standard*” serological test for visceral leishmaniasis is IFA^11^, with better accuracy in non-endemic areas, however this is highly questionable^30^. In this study, the concordance (modified Kappa) of IFA with DPP^®^ and DPP^®^+ELISA were high (83.9% and 92.9%, respectively), but the proportion of positive concordance was extremely low. 

In a scenario of a non-endemic area, the results led to the suspicion of false-positive results of serological tests, reinforced by 63.2% of the seropositive samples that were negative in the molecular diagnostic test. However, we were not able to completely prove this idea because not all seropositive samples were tested by PCR.

A less likely hypothesis, since the study area is considered non-endemic, is that the seropositive animals could be individuals under conditions of immunological resistance, that is, with low or no positive serology and fluctuating titers, and with a difficult parasite isolation. Resistant infected dogs may remain this way indefinitely or under other concomitant factors that cause the loss of cellular immunity^31^. In addition, serology represents an indirect test that can be positive up to two years after symptomatic infection^31^, even in the absence of the parasite. However, animals with symptomatic VL commonly recover from the disease^32^, even under treatment^33^ ^,^ ^34^. In addition, the positive serology for leishmaniasis is directly associated with parasite presence^35^, and the level of antibodies is directly associated with the parasite load and intensity of symptoms^36^. 

The hypothesis of seropositive animals being dogs with maternal antibodies was discarded, since all animals were over three months old, an age at which there are no more external antibodies^37^. 

It is important to point out that although the region is not endemic for VL, the vector species for *L. infantum* transmission, such as *Pi. fischeri* was and *Mg. migoney* were reported at least 40 km around the area^38^, with the last one was specifically in the continuous forest of CBSP^38^.

According to Reis et al. (2010), low parasite load is directly related to low antibody titers, resulting in a false-negative molecular diagnostic^37^. Therefore, the negative parasitological diagnosis of all seropositive animals could be the true result. This problem is enhanced by the fact that parasites are not homogenously distributed in the tissues^39^. We did not evaluate both LN and CS for all seropositive samples, only one of them for each dog. Thus, the absence of the parasite in LN samples does not predict the absence of the parasite in CS scraping, and vice-versa^12^.

 In general, the diagnostic methods for canine VL should be progressively reviewed, as there is still no truly accurate test for diagnosing asymptomatic dogs in non-endemic areas^21^. Concerning the geographical dispersion of VL, the report of human cases occurs after vector presence and after infection in dogs^3^. Therefore, better identification of infected dogs is important for disease surveillance and control. 

In this study, serological and molecular diagnostic results of VL in dogs living in non-endemic areas were described. Although there was 2.3% positivity for DPP^®^+ELISA and 4.7% for IFA, these results did not agree with the molecular analysis in 63.2% of the samples. The quantitative serological diagnostics presented low titers and there was high concordance in the comparison of IFA and DPP^®^ results, as well as in the IFA and DPP^®^+ELISA results. Therefore, we conclude that in non-endemic areas, the VL canine diagnosis should be carefully evaluated, and the serological results should be confirmed with PCR, to avoid false results, as recommended by VLCSP.
