# Supplementary figures and images for: Investigation of canine visceral leishmaniasis in a non-endemic area in Brazil and the comparison of serological and molecular diagnostic tests
Source: Rev Soc Bras Med Trop. 2021 Sep 6;54:e0182-2021. doi: 10.1590/0037-8682-0182-2021 (PMC8437447; doi:10.1590/0037-8682-0182-2021)

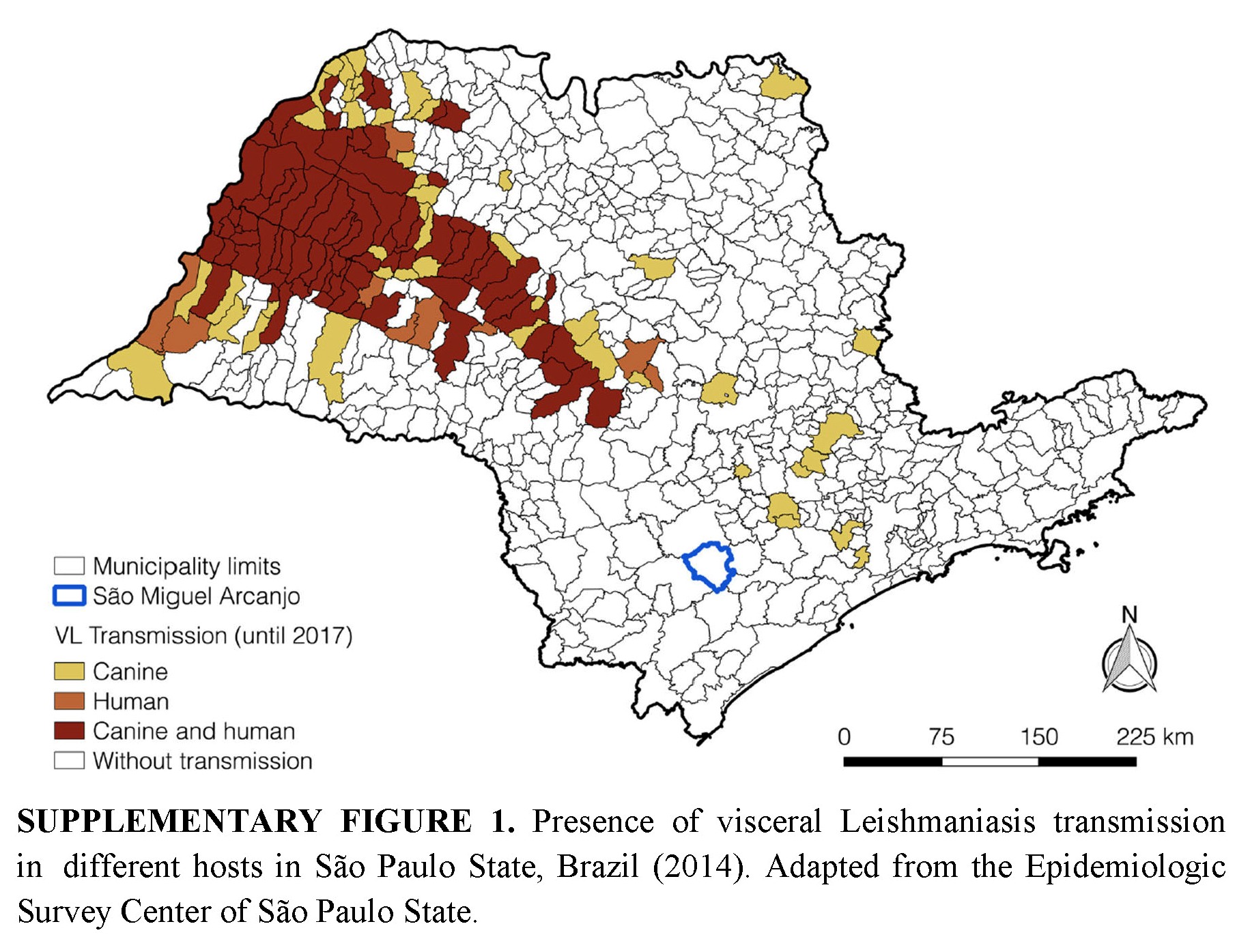

Supplement: Supplementary file 1 [file 1678-9849-rsbmt-54-e0182-2021-supp1.jpg]

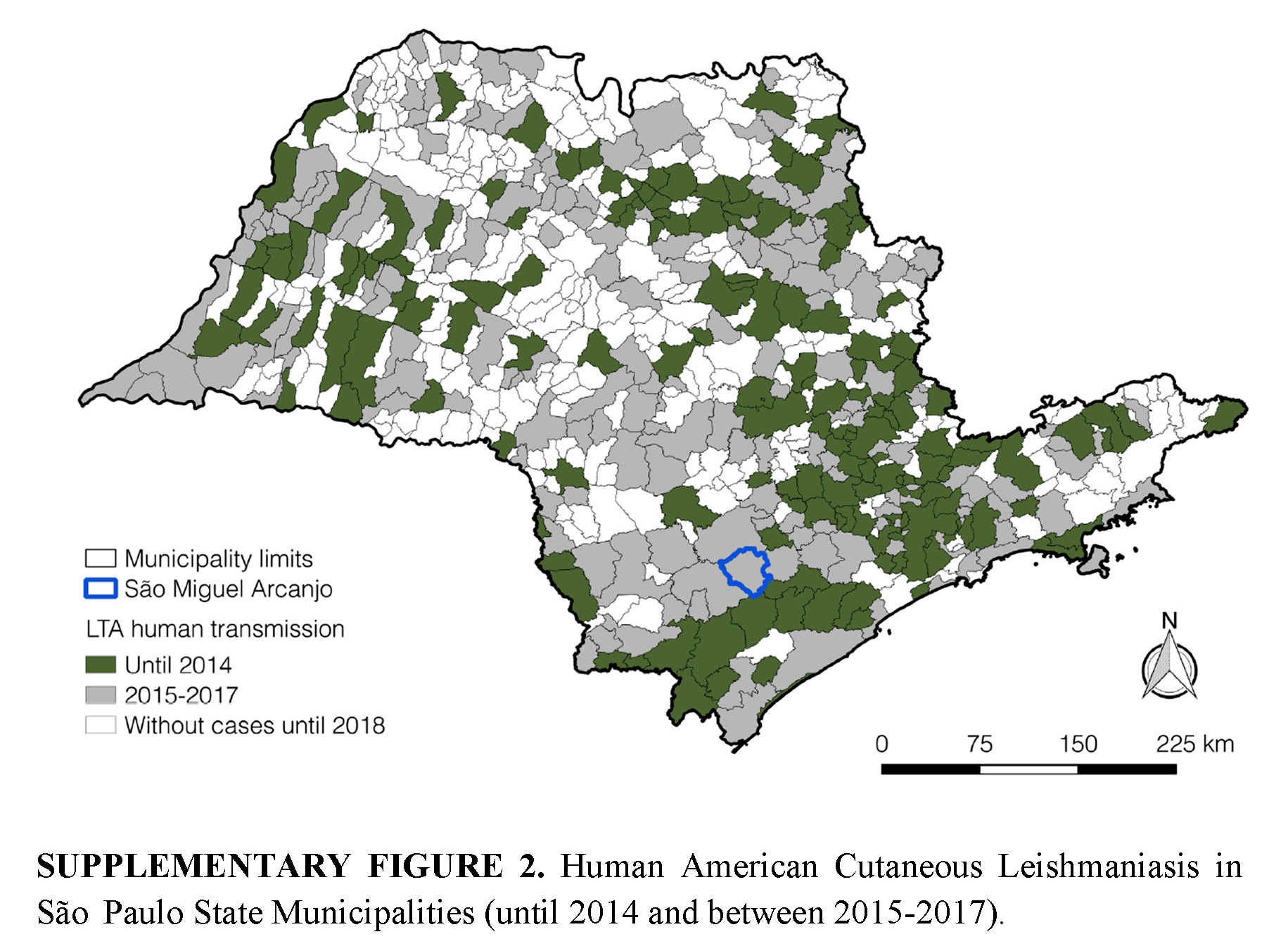

Supplement: Supplementary file 2 [file 1678-9849-rsbmt-54-e0182-2021-supp2.jpg]
